# Local perceptions do not follow rainfall trends: A case study in traditional Marajo island communities (eastern para state, BR)

**DOI:** 10.1016/j.heliyon.2023.e15497

**Published:** 2023-04-17

**Authors:** Davison M.S. Assis, Vânia S. Franco, Thaiane S.S. Dias, Giordani R.C. Sodré, Ana C.C. Tavares-Martins, Bruno S. Godoy

**Affiliations:** a Environmental Science Graduate Program, Federal University of Pará, Belém, Brazil; b Meteorologist at the Institute of Geosciences / Faculty of Meteorology, Federal University of Pará, Belém, Brazil; c Center for Natural Sciences and Technology, State University of Pará, Belém, Brazil; d Aquatic Ecology and Fishery Centre, Federal University of Pará, Belém, Brazil

**Keywords:** Climate perception, Amazon, Coastal zones, Marine Extractive Reserve of Soure

## Abstract

The great current challenge for the conservation and use of natural resources refers to global climate change, because of its impacts felt in different intensities at global, regional, and local spatial scales. Within the system of environmental protection areas in Brazil, the extractive reserves ensure the sustainable use of natural resources by traditional populations, thus maintaining the cultural and biological aspects of a region. Such populations, being in close management of the surrounding environments, tend to perceive changes in ecological processes that many need for their livelihoods. The use of this perception of local populations in conjunction with academic research evidence has a high potential to allow a whole and systemic view of possible changes in natural phenomena. This study developed an integrated analysis of scientific evidence and local perceptions to understand the variation of precipitation in a community inserted in an extractive reserve in the eastern Amazon. We used 30 years of precipitation data from the Brazilian National Institute of Meteorology - INMET, the Southern Oscillation Index - SOI, and the Atlantic Meridional Mode Index - AMM. Furthermore, we applied a form to measure the population's perception of possible changes in rainfall cycles in the region. The meteorological data indicate that the region of the community has been presenting a rainfall reduction; however, people in the community do not perceive this trend. Although it is public knowledge that the global climate is undergoing changes, a fact noted after the integrating analysis of scientific evidence with local knowledge in Resexmar Soure is that the perceptions of traditional populations often focus on smaller temporal and spatial scale visions.

## Highlights

• The annual precipitation rate has decreased over 30 years in Resexmar Soure.
• Local perceptions do not follow the same temporal pattern as those of the scientific evidence.
• Local perceptions are more focused on short time scales (monthly).
• Future studies combining scientific data and local perceptions should work considering short time scales.

## 1 Introduction

Human activities such as burning fossil fuels and deforestation are related to the considerable increase in greenhouse gases in the earth's atmosphere, contributing to climate change [1]. A direct consequence of this scenario is that low-lying coastal areas in Central and South America are increasingly exposed to climate-related risks, particularly due to temperature-increasing phenomena, South El Niño Oscillation (SENO), and storms [2].

Considering this scenario of changes, the projections of the Intergovernmental Panel on Climate Change (IPCC) estimate an average global increase in sea level, which is accelerating and can increase from 0.29 to 1.10 m until 2100 [3]. Given this scenario, coastal environments are the most vulnerable and, consequently, most affected areas [4] by this event, and may undergo changes in their current configuration [5], resulting in degradation and, in extreme cases, its disappearance [4]. Although climate change risk projections for coastal areas are dated to the future [[6], [7], [8]], and are among the main threats to the security of human populations established in these areas [9], their current effects need to be measured. In this context, studies show considerable increases in average air temperature over 30 years [10]. These changes are directly associated with the decline of forest vegetation [11], compromising the populations of Extractive Reserves (Resex) in the Amazon that depend on these resources [12].

Different of the increasing number of studies to understand the phenomenon and its negative effects, the local knowledge of populations had a little attention of the science [13,14]. Considering man as an integral part of nature, we understand that the social approach to understanding the phenomenon is as relevant topic. Perception consists of how human beings see the environment and how they understand the laws that govern it, this view is the result of knowledge, experiences, beliefs, emotions, culture and actions, translating into experiences [15]. It is inherent to each person, based on how they perceive, react and respond, both to interpersonal relationships and actions in the environment [16]. Considering that the reaction of individuals is part of their interpretation of a given event [17], the understanding of the environmental perceptions of coastal populations and the factors that influence them can provide important information to communicators about threats in the coastal environment, while helping to clarify the influences on general perceptions of climate-related risks [18].

Climate has received much importance in scientific studies, employing various approaches and as a result different trends of precipitation and temperature have been found [[19], [20], [21], [22], [23]]. However, it can be seen that studies that jointly evaluate data from meteorological variables and local perceptions have been advancing timidly, requiring a greater effort by the scientific community to overcome this gap [[24], [25], [26], [27]].

The congruency of social perception and scientific data enables a more effective communication between academia and society, influencing the acceptability of local communities in relation to possible interventions and/or public policies for mitigation and adaptation to climate change [28,29]. However, this congruency isn’t a currently pattern, resulting in a divergence between the subjective evaluations of the residents and the objective data on the dynamics of changes in certain environmental elements [30,31]. To test that local communities had an environmental perception in congruency with the scientific data, we studied the social knowledge of three coastal communities in eastern Amazonia about changes in rainfall patterns.

The coastal communities covered by this study are inserted in the area delimited by the Marine Extractive Reserve of Soure (Resexmar Soure) which is located in a coastal region, being therefore more exposed and vulnerable to variations in weather patterns, according to current forecasts. These variations may compromise the well-being of the local population that uses the preservation area of Resexmar Soure for the exploitation of natural resources and marine fishing, activities that make up the livelihood and complement their family income. Based on these considerations and based on the characteristics of the region, the following hypotheses were tested: 1 - The population of Resexmar Soure perceives changes in local rainfall patterns recorded through meteorological stations; 2 - Changes in these patterns have affected the well-being of the local population. To answer these hypotheses, precipitation and individual perception data were collected from meteorological indices from the application of forms to residents, which were analyzed from the geographic perspective of perception.

## 2 Material and methods

### 2.1 Characterization of the study area

**Fig. 1:**
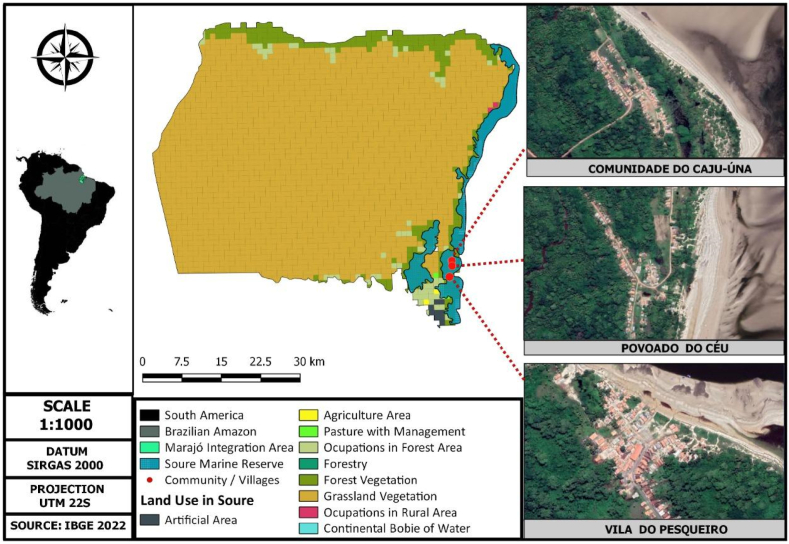
Location of Resexmar Soure and land use and land cover in its vicinity.

Resexmar Soure is a Conservation Unit located in the coastal region of Marajó Island, bordered to the north by the Atlantic Ocean, to the south by the municipality of Salvaterra, to the east by the Marajó Bay, and to the west by the municipalities of Chaves and Cachoeira do Arari [32] (Fig. 1). The Resexmar Soure is composed of three communities, which are respectively distant from the urban center of Soure: Vila do Pesqueiro, 7 km; Comunidade do Caju-Úna, 18 km; and Povoado do Céu, 23 km [33].

In the region the average annual precipitation is greater than 3000 mm. year^−1^ [34], and the period of December occurs the greatest accumulation of precipitation [> 400 mm; 41]. The relative humidity during this period is 84% and the air temperature varies between 23.3 °C and 31.5 °C. Between June and November, the average monthly precipitation is less than 105.0 mm, with a relative humidity of 78% and temperatures between 24 °C and 32 °C [35]. Thus, it is characterized by a typical rainy and humid climate regime with relatively low air temperatures in the first half of the year and, conversely, a dry regime with relatively high air temperatures in the second half of the year [36].

The primary source of income for traditional human populations is the use of natural resources, in an orderly manner and with little predatory potential, in addition to wages for civil servants and retired people, as well as the payment of government benefits for traditional communities, such as Seguro Defeso, Bolsa Verde and Bolsa Família. The main extractive activities are fishing, crab and shrimp harvesting, and extraction of forest products [33]. These populations coexist and develop themselves based on the knowledge transmitted by their ancestors or their experiences in the field [37]. The target audience of this study, the residents of the Resexmar Soure, is located in natural environments, whose vegetation is composed of restinga areas [38], dry and flooded fields, as well as *tesos*, which are areas of anthropogenic origin formed during the occupation of the island by pre-Columbian populations [39].

The community members build their houses on wooden pillars (palafitas type) to prevent them from flooding and being carried away by the current, which is stronger between the months of February and March. They built their houses with federal government funds in partnership with the National Institute for Colonization and Agrarian Reform (INCRA) [33]. The houses have electricity, piped water, and some have internet access. Each community has a primary school, a meeting center for cultural and religious events, and a primary care clinic that treats common illnesses such as flu, cold, headaches, gastrointestinal problems, and injuries [40].

### 2.2 Data acquisition

#### 2.2.1 Meteorological data

We used data on precipitation from the Brazilian National Institute of Meteorology (INMET), from the Soure station, located at −0.73 latitude and −48.52 longitude and 12.6 m altitude, for the period from 1989 to 2019 (31 years). The Climate Prediction Center/National Oceanic and Atmospheric Administration (CPC/NOAA) provided Southern Oscillation Index - SOI and Atlantic Meridional Mode - AMM index data to characterize precipitation variability. These data provide monthly Sea Surface Temperature (SST) and Mean Sea Level Pressure (MSLP) anomaly values for the period January 1989 to December 2019, with a total of 372 values, for both the Atlantic (AMM) and Pacific (SOI) Oceans.

#### 2.2.2 Processing of the meteorological data

Calculation of the standard deviation of the 31 years of rainfall data provided by INMET, determined the range of precipitation variability [41]. In this sense, the most expressive deviation results considered are those above or below the established variability range.

The Northeast of Pará annual climatology, where the Marajó mesoregion is located, is related to the large-scale influence systems such as the ITCZ (Intertropical Convergence Zone), strong local convection, Cumulunimbus agglomeration and because of the location near coastal areas, to the squall lines [42]. Large-scale systems are influenced by planetary ocean-atmospheric mechanisms, such as the Atlantic dipole (showing opposite signs of anomalies to the equator north and south) and El Niño-Southern Oscillation (ENSO). These mechanisms contribute to changes in the atmospheric circulation that are responsible for interannual precipitation variability over the Amazonian northeast. As a result, it was decided to use the Southern Oscillation Index (SOI) and Atlantic Meridional Mode (AMM) index since the phenomena to which these indices refer (El Niño, La Niña, and Atlantic Dipole, respectively) directly affect the interannual variability of precipitation.

The Southern Oscillation Index is a numerical development and intensity indication of the El Niño Southern Oscillation (ENSO), and it is calculated using the average pressure differences at sea level/atmospheric pressure between Tahiti and Darwin based on a monthly analysis in order to define the high and low SOI values, related to the phases: cold.

(La Niña, SOI+) and hot (El Niño, SOI-) from ENSO [43]. The Atlantic Meridional Mode (AMM) is calculated based on the Sea Surface Temperature (SST) that shows the interhemispheric gradient performance (Atlantic Dipole). The AMM does not contribute to the rain formation at its positive phase and contributes to rain formation within the region due to ITZ north-south modulation over the ocean at its negative phase [44].

We also chose to apply the nonparametric Mann-Kendall test [45] in the time series of precipitations from Soure station between 1989 and 2018 (30 years) where the monthly precipitation values were added to generate annual precipitation (PRP) and seasonal rainy regime (December to May) and Dry regime (June to November) - to detect positive/negative - or increase/decrease - significant trends, according to Refs. [[46], [47], [48]].

### 2.3 Obtaining the social data

#### 2.3.1 Sampling

Resident informers were selected from one of the communities within Resexmar Soure, with ages equal to or over 18 years old. In total 112 interviews were made with 46 in Vila do Pesqueiro, 31 in Comunidade do Caju-Úna and 35 in Povoado do Céu (Table 1). This number reflects the maximum sample effort obtained in the data collection and, according to the last survey [49], corresponds to 44.98% of these communities' population counted 249 families. Our sample presents a 93% confidence index which is an acceptable rate for human population research [50].

**Table 1:** Socioeconomic data of respondents in Resexmar Soure.

Gender	Nº	%
Male	56	50.00
Female	56	50.00
**Age**		
18 to 20	8	7.14
21 to 40	26	23.21
41 to 60	42	37.50
61 or more	36	32.14
**Education level**		
Incomplete elementary school	60	53.57
Complete elementary school	7	6.25
Incomplete high school	10	8.92
Full high school	28	25.00
Complete higher education	7	6.25
**Length of residence**		
1 to 10	14	12.50
11 to 20	20	17.86
21 to 40	21	18.75
41 or more	57	50.89
**Household size**		
1 to 3	58	51.79
4 to 6	44	39.29
7 or more	10	8.93

#### 2.3.2 Preparation of the perception forms′

A two-section structured form (supplementary material 1) served to obtain the data. The first section was: I. Socioeconomics, and comprise variables such as: i. gender; ii. age; iii. years of residence; iv. annual income; v. number of people in the household; and vi. years of schooling. The second was: II. Perception of precipitation in the community. The levels of perception in section II had assertive characteristics, on a 5-point Likert scale (where 2 means strongly disagree, and 10 means strongly agree) [51]. By answering the assertions, respondents indicate their degree of agreement regarding a situation or scenario presented. This tool has been widely used to understand perceptions of climate change [[52], [53], [54]].

The framing of the assertions considered the context of changes in rainfall patterns in order to understand how the residents of Vila do Pesqueiro perceived this phenomenon. In this sense, considering the empirical knowledge of traditional communities, the community members contributed by answering how often they have perceived changes in rainfall patterns, and whether this has affected their local well-being.

#### 2.3.3 Assessment of the agreement of changes in local rainfall level and well-being

The development of an agreement scale based on the work of Assis et al. (2020) [40] made it possible to quantify the degree of agreement on perceived changes in local precipitation patterns. The quantification of the assertions derived from the answers in the forms indicated the level of agreement of the respondents in relation to the phenomenon presented. The agreement scale, which assessed the degree of agreement, ranged from 2 to 10, considering 2 as the minimum score scale (no agreement) and 10 as the maximum score scale (very high agreement, Table 2).

**Table 2:** Classification of levels of perceived changes (Adapted from Assis et al. (2020).

Classification	Score
No agreement	2.0
Low agreement	2.1 to 4.0
Agreement	4.1 to 6.0
High agreement	6.1 to 8.0
Very high agreement	8.1 to 10.0

To assess the well-being of community members, we considered Kahneman's approach [55] that proposes five conceptual levels for well-being research: 1. External conditions (e.g., income, neighborhood, housing); 2. Subjective well-being (e.g., self-reports of satisfaction and dissatisfaction); 3. Persistent mood level (e.g., optimism/pessimism); 4. Transient emotional states, immediate pleasures or pains (e.g., joy, anger); and 5. Biochemical and neural bases of behavior. We chose to use "subjective well-being," to capture the degree to which respondents are satisfied or dissatisfied with possible perceived changes in rainfall patterns. Such an approach focuses on people's subjective judgments and experiences [56].

To complement the analysis of perceptions, we adopted a field diary to record the events relating to the days of interviews, as well as people's understandings, transcribed in speeches, about the phenomenon studied and their relationships with nature [57]. The descriptions reported by the community members were interpreted based on Content Analysis [58], which aims to extract expressed or latent meanings from a message. In this type of analysis, the researcher seeks to penetrate the ideas, mindsets, values, and intentions of the communication producer to understand his message. The content is analyzed from the sociocultural context of the message producer: the intentions, pressures, conjuncture, and ideology that conditioned the production of the message in an effort to articulate the objective, quantitative rigor with comprehensive, qualitative richness.

The analysis of the socioeconomic data of the residents applied descriptive statistics with the measurement of the percentage in the different categories, namely: age, years of residence, education, and household size. The analyses were conducted in R software [59], presenting the data in a table (Table 1).

## 3 Results

### 3.1 Annual precipitation and the SOI and AMM indices

**Fig. 2:**
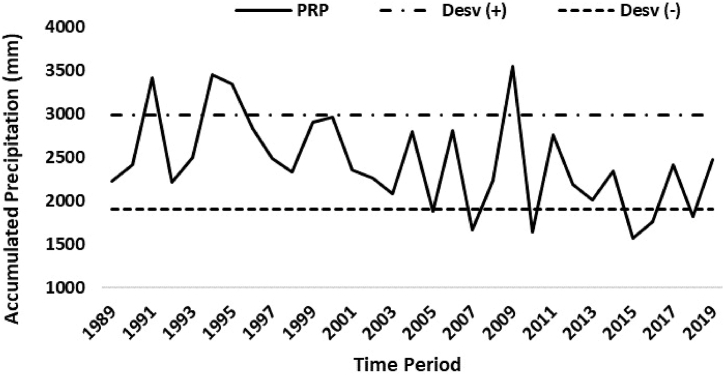
Annual precipitation series, from Soure station for the period 1989 to 2019, where PRP = precipitation; Desv (+) = Standard deviation above the mean; Desv (−) = Standard deviation below the mean.

The region's precipitation varied in the analyzed period between 1500 mm and approximately 3500 mm annually, according to INMET data (Fig. 2). Precipitation on an annual scale and in the rainy season showed a tendency to decrease in the observed period (Table 3). There is a marked variability of annual rainfall in this period, with highlights for some years that reached expressive rainfall values, as in the cases of 1991, 1994, 1995, and 2009, in which values close to 3500 mm of annual rainfall are noted. The variability of annual precipitation shows a pattern within normality, except for the years 1991, 1994, 1995, and 2009, when there were episodes of occurrence of rainfall above the normal pattern (anomaly), indicating that the respective years were very rainy. On the other hand, anomalies also occurred in the years 2007, 2010, 2015, and 2016. However, these were years the rainfall volume was below the expected standard. Such variation observed in that precipitation might be associated with the ocean-atmospheric phenomena that act in both the Pacific (El Niño and La Niña) and Atlantic (Atlantic Dipole) Oceans.

**Table 3:** Annual precipitation (PRP) and Seasonal trend - seasonal rainy regime (December to May) and Dry regime (June to November) at Soure station based on Mann-Kendall test during 30 years, between 1989 and 2019.

Variables	Z	S	tau
PRP_annual	**−2.72**	**−161**	**−0.35**
Rainy	**−2.75**	**−163**	**−0.35**
Dry	−1.24	−74	−0.16

Z is the Mann-Kendall parameterized statistical test, S is the score of Mann-Kendall and tau is correlation coefficient value to Mann-Kendall. The values in bold indicate P < 0.01.

We observed the balance of occurrences of rainfall extremes (pattern outside the range of rainfall - in dashed lines), with four years of the occurrence of high rainfall volume and four years of low rainfall volume. In addition, most of the events of high rainfall occurred in the 1990 decade, with the exception of only the year 2009, while the years that presented low rainfall volume, became frequent from the year 2007, and started to occur at intervals ranging from three to five years.

**Fig. 3:**
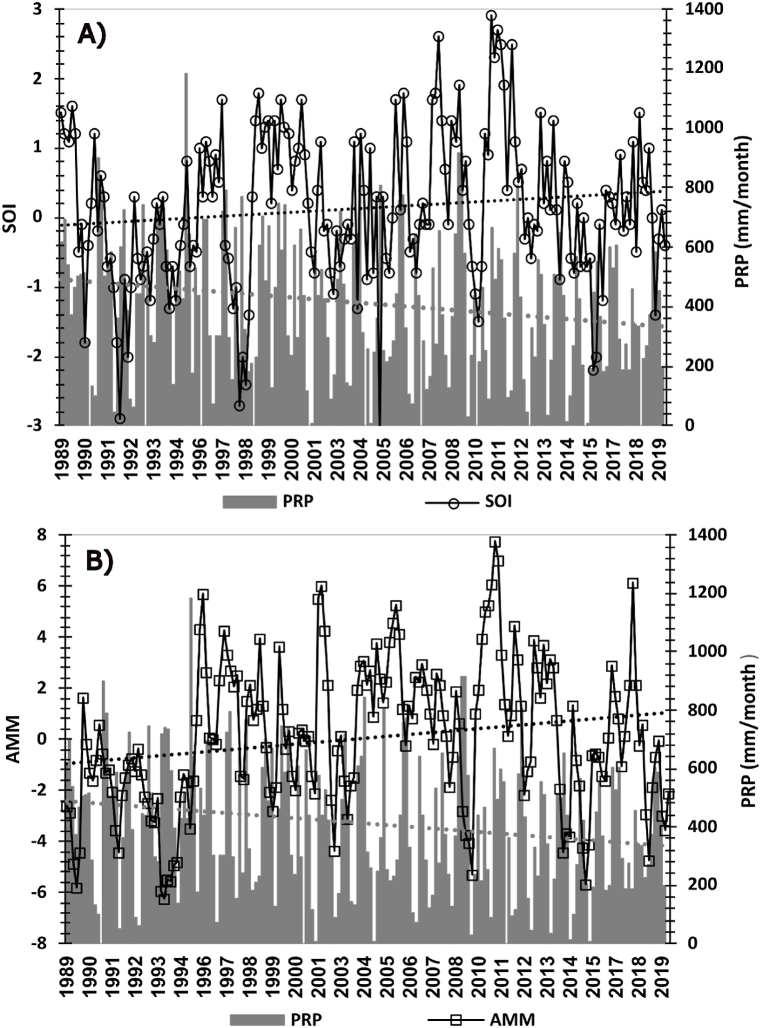
Monthly precipitation series (PRP) and SOI(A) and AMM(B) index for periods between 1989 and 2019. The dashed lines show the index and precipitations tendency.

A small increase in tendency is observed in the SOI positive phase (La Niña, Fig. 3a), which suggests a pluviometry increase. However, it disagrees with the data presented for the INMET Soure station (Fig. 2, Fig. 3) since it exhibits a negative rain tendency, especially in the last decade. Moreover, there is an accentuated tendency in the positive AMM phase (Fig. 3b) corroborating with the precipitation information from the analyzed period. In that way, we consider that the Soure precipitation during the research period is under a greater influence of the phenomenon that occurs in the Atlantic (Atlantic Dipole), as it influences Intertropical Convergence Zone oscillation (north-south), contributing directly when it is positioned further south, in the precipitation of the Amazon in austral autumn (March, April, and May).

### 3.2 Perception in Resexmar Soure

#### 3.2.1 Socio-economic characterization and rainfall perception of the interviewees

**Fig. 4:**
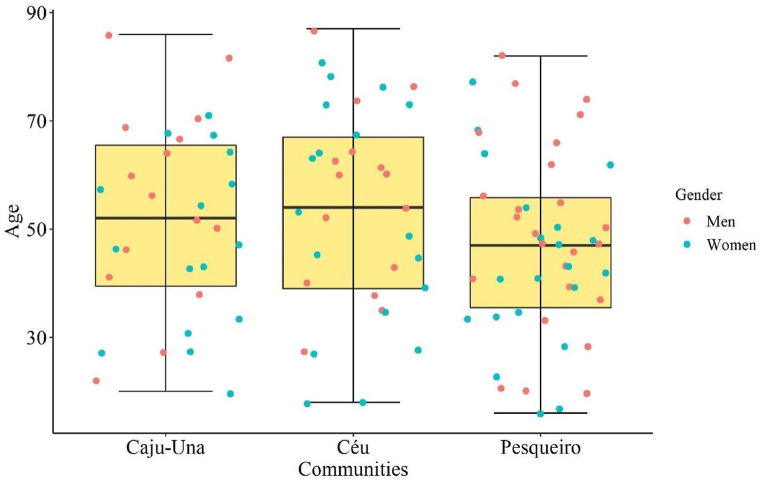
Age distribution in the three communities.

The majority of that population is within an older age group between 41 and 60 years old, and the younger minority is in an 18 to 20 age group, and the distribution of age was similar on the three communities (Fig. 4). It is also noted that the majority have an incomplete basic education level and is less than where capable of concluding the basic education level. Concerning residence time, the age pattern is repeated. The majority of the population lives is living in the same community for a period longer than 41 years. As for the size of the household, it is noted that families are largely composed of 1–3 people in the household.

Local perception revealed that community members mostly believe rain has become more amounts in the community (7.80) (Table 4). This finding is endorsed by a low concordance level on the statement that addresses the low amounts of rainfall (2.74) and the low level of agreement in the assertion that address the stability of rainfall patterns in communities. Community members do not relate recognized changes in the rainfall pattern to the population damage of Resexmar Soure, as the level of agreement for such a statement is low (3.47). The reported consequence of increased rainfall in the region is the population's illness (6.50), as evidenced in the speech of some community members below.

“With rainfall increase, diseases such as the flu appear”

NASCIMENTO, L., 54 years old.

“With climate change, the most common diseases in the community always appear, such as cough and diarrhea” SANTOS, R. M.N., 54 years old.

**Table 4:** Level of perception about precipitation in Resexmar of Soure.

Assertives	Perception level scores
1 – It started to rain more in the community	7.80
2 – It started to rain less in the community	2.74
3 – Rainfall patterns remain the same since I have been established in this community	3.07
4 – I haven't been able to notice changes in rainfall patterns since I've been established in this community	3.27
5 –The decrease rainfall started to harm community residents	2.18
6 –The Increased rainfall started to harm community residents	3.47
7 – The People in the community became more ill due to changes in rainfall patterns	6.50
8 - Community residents began to complain more about climate change	7.72

It is relevant to highlight that when residents use speech to refer to the term “climate change” they are referring to changes between dry and rainy periods. In this sense, based on the statements results and on the speeches of the community members, it is observed that the reported increase in the amounts of rain does not present significant damages that compromise the community’s well-being since they offer access to a health center that facilitates treatment, in addition to traditional knowledge that, through the use of medicinal plants, provide alternative medical care that helps treat and restore these illnesses.

The speech of AMARAL, L., aged 35, resident of Caju-Úna, highlights the ability to adapt to changes in the sentence below:

“The Community residents adapt over time to climate changes AMARAL, L., 35 years old.

It is noticed this belief is common among communitarians since they believe that because they are exposed to the alterations, they will be capable of developing adaptative mechanisms in order to reestablish themselves from the illness. This result reaffirms once again the communitarians' perception that the rain pattern observed changes do not generate consequences that constitute severe threats to the well-being of communities.

As a general rule, they also agree that community residents began to complain more about climate change (7.72), believing that its effects can already be seen in the gradual loss of coastal environment in certain areas, which has been occurring in Resexmar Soure over the years. This perception can be reiterated in the speech of SALES, S., 64 years old, Povoado do Céu resident:

“With the beach rise, I lost my old house and had to rebuild it in another part of the community” SALES, S., 64 years old.

Besides the natural loss, which is a natural dynamic of the local coastal environment, the residents attribute the acceleration of this process to human action in nature - the main cause of climate change - as NASCIMENTO, A., 52 years old, a resident of Vila do Pesqueiro, claims:

“Nature doesn't mess with anyone, we mess with it” NASCIMENTO, A., 52 years old.

Thus, we note that the perceptions of residents about changes in rainfall patterns, as well as how this can affect the collective well-being in Resexmar Soure, are based on their observations about the phenomenon. Besides that, we also notice that traditional knowledge is present in the construction of these perceptions, which are evidenced in the speeches. This knowledge shapes their worldview, governs their actions, and characterizes the traditional Amazonian lifestyle.

## 4 Discussion

The coastal area of Marajó, where Resexmar Soure is located, has undergone changes due to changes in local precipitation patterns over a 30-year period (1989–2018). Considering this scenario, perceptions reveal that community members have noticed changes, but these do not follow the downward trend demonstrated by the weather station data, which the MMA index corroborates. Furthermore, the perceptions also reveal that the perceived changes do not affect the well-being of communities.

The precipitation results in this study are in agreement with others that have used different indices and analyses to assess changes in weather patterns in the region, such as precipitation estimated by the Tropical Rainfall Measuring Mission (TRMM) satellite [60] and the use of indicators of climate extremes, the Standardized Precipitation Index (SPI) [61]. These observed trends of decreased precipitation may lead to disasters associated with droughts, as highlighted by other studies [60,62]. As for the causes of its variability, they can be attributed to the dominant patterns in the Pacific and Atlantic oceans, which are associated with the occurrence of rainfall in the Eastern Amazon (location of the area of this study), such as the ENSO phenomenon (El Niño and La Niña) and the Atlantic Dipole [63], thus having a direct relationship with the increase or decrease of rainfall in the Amazon.

As perceived in the monthly series of the SOI and AMM indices, the positive trend of the MMA is in agreement with the negative trend of the variability of precipitation at the Soure station, evidencing the interrelationship between the variability of precipitation and the sea surface temperature of the Atlantic Ocean. In this regard, Limberger and Silva [64] showed the importance that the Atlantic has in modulating rainfall in the Amazon basin, which occurs by the entry of moisture by the trade winds such as the positioning of the ITCZ.

Furthermore, as anomalously warm waters in the tropical North Atlantic define the positive AMM, it results in decreased rainfall for the rainy season in the Amazon basin, due to the weakening of the northeast trade winds, which causes the ITCZ to position further north than normal. In addition, according to the AMM index data, this has been occurring frequently in recent years. During the positive AMM, the moisture flux divergence is less negative in the austral summer months (when the rainy season starts in the Amazon), indicating less moisture flux to the region in these periods [65].

As for the fact that the community members believe that rainfall has become more frequent in the region, this is due to their perceptions being centered on observations made over the last few years, in which the community members have noticed a high amounts of rainfall in periods considered unusual for the region. One can understand this fact better from the speech of some members of the Vila do Pesqueiro:

“The rains have become more frequent in the community; it is raining out of season” RIBEIRO, C., 50 years old.

“It was not common to have rains in the months of July and August; in times past, the rains would have already ceased” CARNEIRO, M. L., 57 years old.

We noticed that the communitarians' perception regarding precipitation is based on shorter periods as months. In this regard, we raise the hypothesis of short temporal distance addressed by the Psychological Distance Theory [66,67]. According to this approach, the longer the time of an event, the more abstract its understanding becomes [68,69]. In the present study, the precipitation analysis follows a meteorological pattern, considering a minimum period of 30 years; for perceptions, these follow a personal interpretation perspective, and that generally presented an understanding pattern within a shorter time scale (months). In this context, Pahl et al. [70] points out that this is due to the human thinking limitation about the future, which hardly exceeds ten years since its social constructions are short and based on 4-year electoral cycles and time horizons of 5–20 years used in community planning.

Importantly, most of the survey participants are older people, fishermen, and direct descendants of the first Resexmar Soure settlers [32], therefore longtime residents within their respective communities. Because they have been established in the region for years and, particularly in a Conservation Unit, they have built a world vision that is little degraded, based on sustainability and environmental balance [40,71]. We believe that this reflects in the way they face global climate change and precipitation patterns since although they perceive such changes, they assume that these events do not constitute threats to the communities, despite current forecasts demonstrating that this is real [2,12].

Although community members believe that changes in the pattern of precipitation and climate change can cause the emergence of some diseases, they do not attribute significant damage to climatic events. Our results reveal that they make use of alternative methods to treat these diseases, with practices that take place through the use of plants to prepare tea, infusion, and baths, in addition to their use in mystical and spiritual rites [49,72] believe this explains the low-risk perception, as from all the knowledge gathered throughout their experiences at Resexmar Soure, they were able to develop an adaptation strategy given this scenario of changes.

A recent study for the Amazon reveals that global climate change is the main factor that alters precipitation [73]. In this sense, our results of the meteorology analysis suggest that Resexmar Soure, being located in the Amazon biome, is facing the results of these changes, even if in small proportions. The perceptions of the communitarians show the same direction of these changes within that context it is important to highlight that community members believe that the world has been experiencing climate change, a viewpoint that follows a common thought pattern among people [74,75].

A study published on the Amazon depicts a common pattern for the entire region, with rainfall occurring more frequently in the months from December to May, and less frequently in the months from July to November [76]. However, based on local perceptions in Resexmar Soure, we noticed that the communities have been experiencing frequent unseasonable rainfall. In this regard, the work of Paca et al. [77] revealed that rainfall trends in the Amazon are not spatially uniform. Thus, by integrating perception and scientific data, it is evident that although the temporal (monthly) patterns of precipitation seem altered in Vila do Pesqueiro, it does not mean to say that the residents' observation is accurate, since their perceptions are tied to changes in the amounts of rainfall in months that were previously uncommon, and not in relation to years.

We believe this is also due to the community members' difficulty in portraying information that takes into account a long period of time, considering that they presented a scenario based on their most recent observations. Although expected, in general, that people portray perceptions according to climate trends [78], it is important to stress that they can vary greatly from individual to individual, and particularly from community to community [79]. Besides, in the case of Resexmar Soure, we noted that residents were unable to indicate a decrease in precipitation patterns as indicated by meteorological data.

## 5 Conclusions

Our study show that local perceptions are not congruent with scientific data, and considering that the longer the phenomenon last, the more abstract its understanding becomes, carrying out future studies based on the Theory of Construction Levels is important for us to understand from which temporal, social, or geographic scales these perceptions are shaped. With regard to perceptions of climate change, a more in-depth study is necessary, given that we carried out a secondary assessment of this phenomenon.

For policy makers and local interveners, the results highlighted in this study are important because they point out that clarification actions about the direction of changes in rainfall patterns are useful for the future. This is necessary, since in any successful adaptation or mitigation strategy, the object to be mitigated needs to be known by the actors involved in the process, generating a certain degree of acceptance. Thus, our results help in directing future actions, considering that drastic changes in rainfall patterns, and even the influence of climate change can have serious consequences for the permanence and survival of communities in Resexmar Soure.

## Author contribution statement

Davison M. S. Assis: Conceived and designed the experiments; Performed the experiments; Analyzed and interpreted the data; Wrote the paper.

Vânia S. Franco; Giordani R. C. Sodré; Thaiane S. S. Dias: Conceived and designed the experiments; Performed the experiments; Contributed analysis tools or data.

Ana C. C. Tavares-Martins; Bruno S. Godoy: Conceived and designed the experiments; Analyzed and interpreted the data; Wrote the paper.

## Data availability statement

The authors do not have permission to share data.

## Ethical aspects

The proposal is part of the doctoral project of the first author and was submitted to the Biodiversity Authorization and Information System (Sistema de Autorização e Informação em Biodiversidade - SISBIO), n 77218-1 and Brazil Platform n: 4.486.124 in order to carry out a research project involving humans.
