# Supplementary material for: Local perceptions do not follow rainfall trends: A case study in traditional Marajo island communities (eastern para state, BR)
Source: Heliyon. 2023 Apr 17;9(4):e15497. doi: 10.1016/j.heliyon.2023.e15497 (PMC10161699; doi:10.1016/j.heliyon.2023.e15497)
Supplement: Multimedia component 1 [file mmc1.docx]

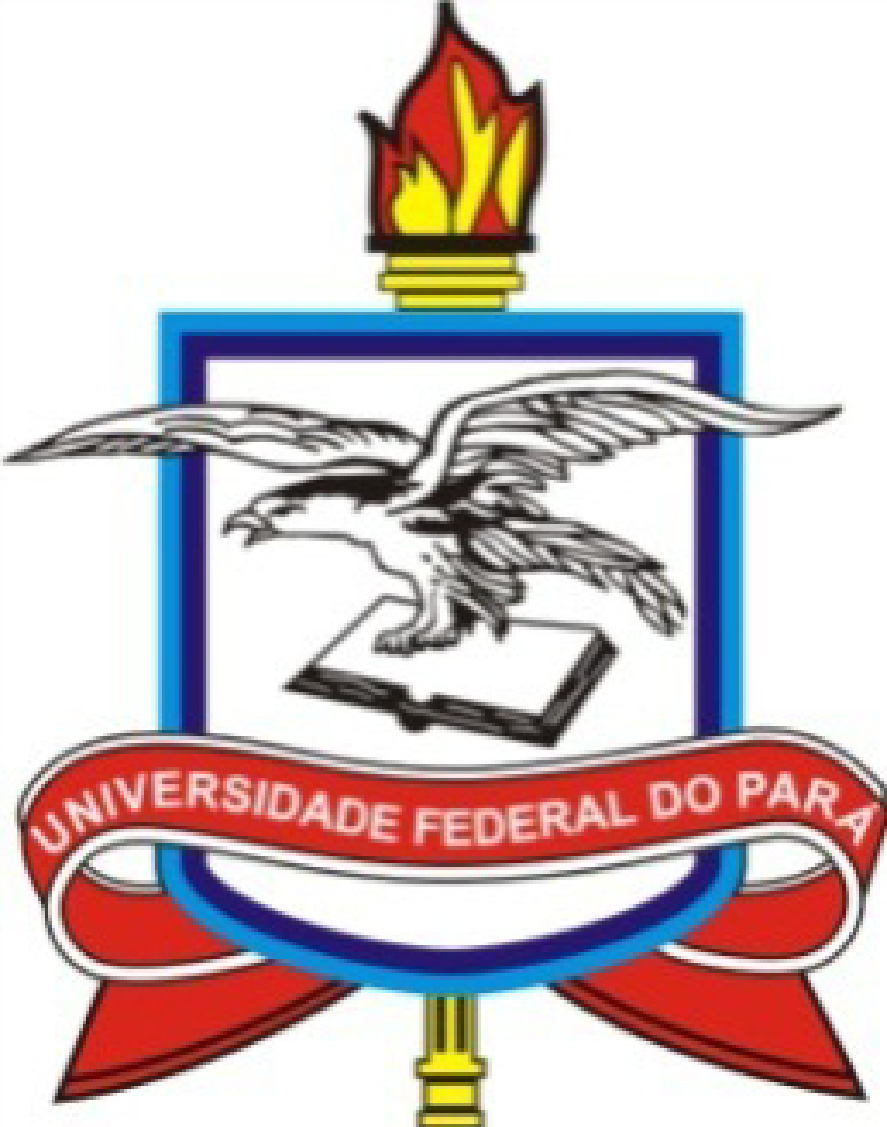


**Supplementary Material 01**

**Form**
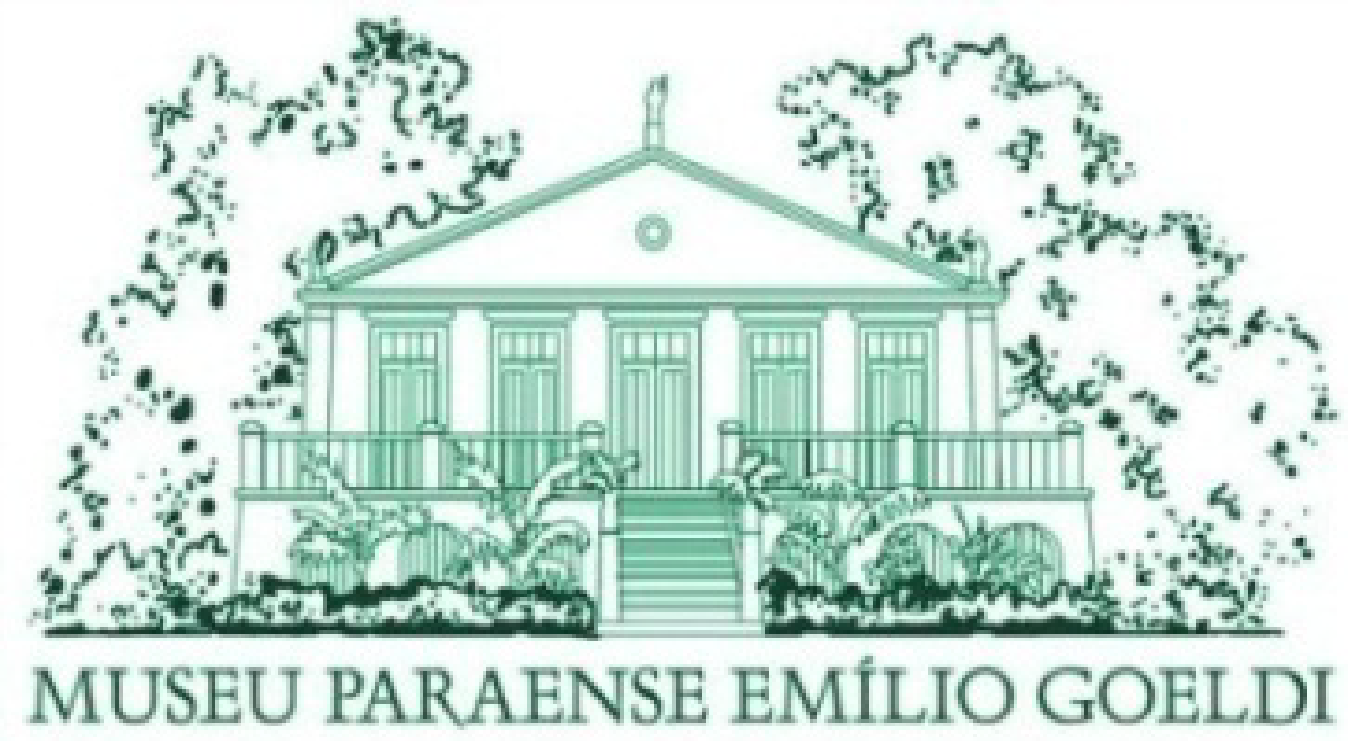

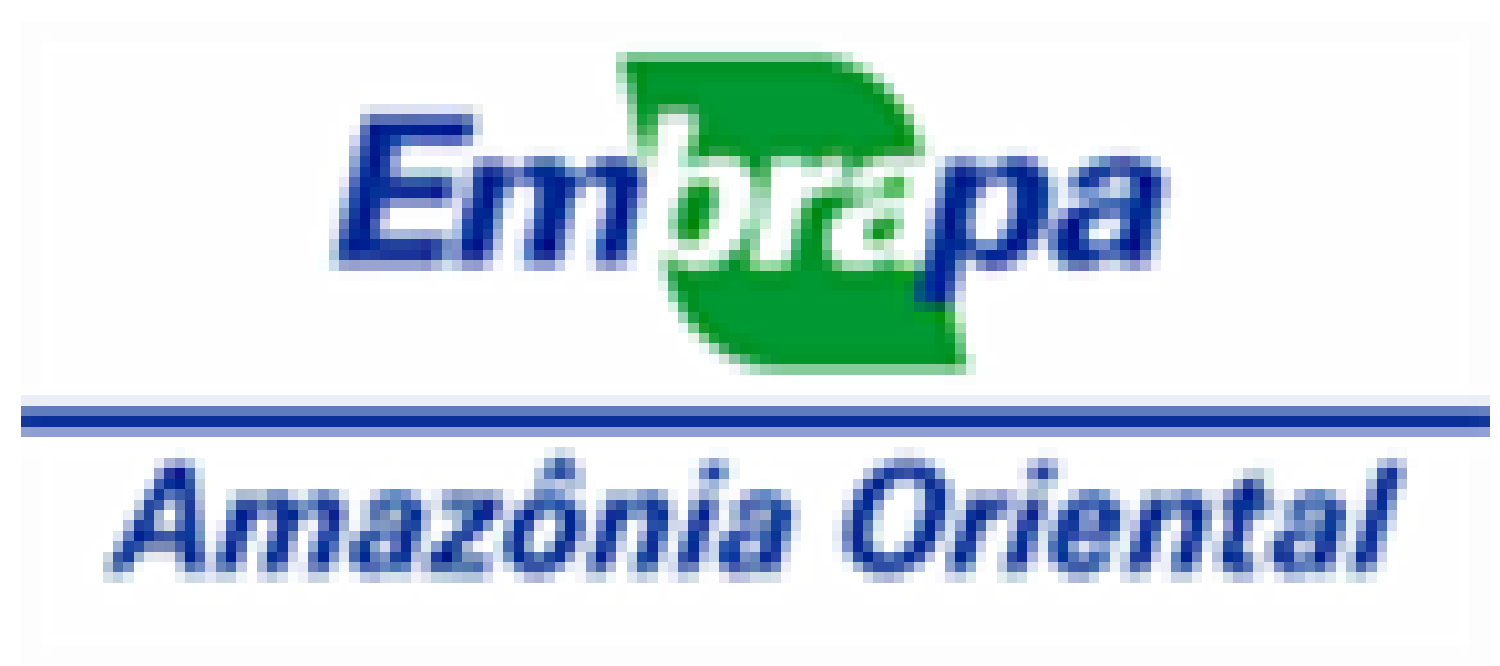


UNIVERSIDADE FEDERAL DO PARÁ - INSTITUTE OF GEOSCIENCES

GRADUATE PROGRAM IN ENVIRONMENTAL SCIENCES

Agreement UFPA/EMBRAPA/MPEG

UFPA/EMBRAPA/MPEG Agreement

1. Socioeconomic Profile

Name of interviewee: _________________________________________________

Interviewee's community: ______________________________________________

Gender of respondent: 1. Male [ ] 2. Female [ ]

Age: _______ Occupation: ______________________

a) How long have you lived in the community?

b) How many people live in your house?

c) What is your monthly income?

d) Until what grade did you study?

e) What is your religion?

1. Christian [ ] 2. Spiritist [ ] 3. Umbanda [ ] 4. other [ ]

Changes in rainfall patterns

According to your opinion, and if you believe in climate change, by what are they caused by:

| **Scoring** | **2** | **4** | **6** | **8** | **10** |
| --- | --- | --- | --- | --- | --- |
| Meaning | Strongly Disagree | Disagree Partially | Indifferent | Partially Agree | Totally Agree |
| It started to rain more in the community | | | | 2 4 6 8 10 | |
| It started to rain less in the community | | | | 2 4 6 8 10 | |
| Rainfall patterns have remained the same since I have been settled in this community | | | | 2 4 6 8 10 | |
| I haven't been able to notice changes in rainfall patterns since I've been established in this community | | | | 2 4 6 8 10 | |
| The decreased rainfall started to harm community residents | | | | 2 4 6 8 10 | |
| The Increased rainfall started to harm community residents | | | | 2 4 6 8 10 | |
| People in the community became more ill due to changes in rainfall patterns | | | | 2 4 6 8 10 | |
| Community residents began to complain more about climate change | | | |  | |
| **OBS.:** | | | | | |
